# Antibiotic prescribing practices in secondary and tertiary hospitals in Shaanxi province, western China, 2013-2015

**DOI:** 10.1371/journal.pone.0207229

**Published:** 2018-12-12

**Authors:** Kangkang Yan, Meiling Xue, Dan Ye, Caijun Yang, Jie Chang, Minghuan Jiang, Mingyue Zhao, Hongli Zhang, Yu Fang

**Affiliations:** 1 Department of Pharmacy Administration and Clinical Pharmacy, School of Pharmacy, Xi’an Jiaotong University, Xi’an, China; 2 Center for Drug Safety and Policy Research, Xi’an Jiaotong University, Xi’an, China; 3 Shaanxi Center for Health Reform and Development Research, Xi’an, China; 4 Department of Pharmacy, Xi’an No. 3 Hospital, Xi’an, China; 5 Department of Drug and Equipment, No. 521 Hospital of Ordnance Industry, Xi’an, China; 6 Department of Pharmacy, Xi’an Central Hospital, Xi’an, China; Natural Environment Research Council, UNITED KINGDOM

## Abstract

**Introduction:**

The irrational use of antibiotics is a major driver of antimicrobial resistance. This study aimed to explore how antibiotics were used in secondary and tertiary hospitals in Shaanxi Province, western China from 2013 to 2015.

**Method:**

A retrospective study was conducted with a sample of 16 hospitals in Shaanxi Province (2 tertiary and 14 secondary hospitals; 8 public and 8 private hospitals) using a stratified random sampling method. All of the macro data from these hospitals from 2013 to 2015 were analyzed. All collected data were double-entered and analyzed using Excel 2007.

**Results:**

The percentage of injectable antibiotic prescriptions was 26.6% of all of the antibiotic prescriptions in the secondary hospitals and 14.2% in the tertiary hospitals. Injectable antibiotic prescriptions in private tertiary hospitals (enter %) were more than two times that of public tertiary hospitals (enter %). In both tertiary and secondary hospitals, the percentage of antibiotic prescriptions for outpatients, emergency patients and inpatients were within the scope of the national standards, while the intensity of antibiotic use was higher than the national standard of 40 DDD/100 bed-days. The prophylactic antibiotic use rate in clean surgery was 40.4% in tertiary hospitals and 60.7% in secondary hospitals, which were both higher than the national standard of 30%. The preventive use rate of antibiotics in private tertiary hospitals (55.00%) was more than two times that of public tertiary hospitals (25.90%), and the rate was also higher in private secondary hospitals (61.50%) than in public secondary hospitals (59.70%).

**Conclusions:**

Substantial antibiotic abuse occurred in the sample Chinese hospitals, especially in secondary hospitals. The government should continue to strengthen the administration of antimicrobial use in hospitals. At the same time, medical professional training and interventions for physicians should be conducted to fundamentally reduce the irrational use of antibiotics.

## Introduction

Antimicrobial resistance (AMR) has gradually become one of the greatest threats to global public health of the 21st century [1–2]. A report on global AMR surveillance by the WHO showed that we are headed toward a “post-antibiotic age” [3]. In the communiqué of the 2016 G20 summit, AMR was specifically mentioned as a serious threat to public health, economic growth, and global economic stability [4].

Increased AMR rates may lead to prolonged hospitalization, a prolonged duration of treatment, increased treatment costs, and increased mortality [5–6]. A report from the Centers for Disease Control and Prevention (CDC) predicted that approximately 2 million illnesses and 23,000 deaths each year are associated with drug-resistant infections in the United States [7]. The spread of AMR is relatively serious in China [8–9]. In 2015, the national detection rate of erythromycin-resistant *Streptococcus pneumoniae* and methicillin-resistant *Staphylococcus aureus* (MRSA) was 35.8% and 91.5%, respectively, according to the surveillance of the national bacterial drug resistance network [10].

The widespread and irrational use of antimicrobial agents is considered the main cause of increased AMR [11–12]. The phenomenon of antibiotic overuse is particularly acute in China compared with other countries [13]. According to a survey of antibiotic use at 48 health care institutions in 2014, the antibiotics prescription rate of hospitalized patients was 77.5%, and only 24.6% of prescriptions were appropriate [14].

To effectively curb AMR and promote the rational use of antimicrobial agents in healthcare settings, the Chinese government launched a national antibiotic stewardship Action Initiative campaign (hereafter referred to as the Action Initiative) in 2011. The Action Initiative aims to control total antibiotic use in hospitals [15]. In China, all hospitals are ranked as tertiary, secondary, or primary health care institutions based on the scale of the hospital (beds), technical level, management level, equipment conditions, scientific research capacity, etc. The target of the Action Initiative includes public secondary and tertiary hospitals.

This campaign protocol clearly defines antibiotic procurement, antimicrobial drug use in hospitalized patients, the outpatient antibiotic prescription rate, the proportion of prophylactic use of antibiotics in clean operations, and so on. A few previous studies evaluated the use of antibiotics in healthcare settings before the campaign [16–17] or assessed the overall impact of the campaign [18]. However, information on the use of antibiotics in secondary and tertiary Chinese hospitals after the campaign is lacking, particularly a comparative study between public and private institutions. This is important because understanding the use of antimicrobial agents in these facilities can provide superior data to support further national policy development.

The aim of this research was to explore how antibiotics were used in secondary and tertiary hospitals in Shaanxi Province, western China between 2013 and 2015 and to compare the use of antibiotics in public and private institutions.

## Methods

### Hospitals

A retrospective study was conducted in tertiary and secondary hospitals in Xi’an, the capital city of Shaanxi Province. Xi’an is one of the largest inland cities in western China, with a population of 8,249,300 and an area of 10,096.81 square kilometers [19]. A multistratified grouping random sampling method was used to identify the target hospitals. First, we found the total number of secondary and tertiary hospitals in Xi’an by searching the official website of the Shaanxi Provincial Health and Family Planning Commission, and we determined the public and private hospital composition ratio of 1:1. At the same time, we also considered the degree of cooperation of the hospitals. We then randomly selected two tertiary hospitals (one public and one private) and 14 secondary hospitals (seven public and seven private) for a total of 16 hospitals as a sample survey of institutions. We chose the same level hospitals with a similar total number of patients and inpatients per year. The inclusion criteria were: (1) general hospital (excluding specialist hospitals); (2) the hospital had complete medical institution macro data for statistical analysis; and (3) the hospital dean agreed to participate in this study.

### Data collection

All medical institution data was collected on a quarterly basis at each selected hospital from January 2013 to December 2015. The information mainly consisted of three parts. Part one included the hospital characteristics. Part two addressed the situation of rational drug use in the hospital. This section contained four prescription indicators, in accordance with WHO/International Network for the Rational Use of Drugs (INRUD) recommendations [20]. Part three was intended to analyze the rational use of antibiotics, including the use of antimicrobial agents among inpatients and the prophylactic use of antimicrobials during clean surgery.

Two investigators were assigned to each medical institution, a graduate student filled out the investigational forms, and a pharmacist reviewed the rationality of the data. All investigators received the same training before the survey. The dean of each hospital gave their verbal consent for participation in this study.

### Main measurements

According to the Guidelines for the Clinical Application of Antibacterial Agents [21], descriptions of the indicators related to rational antibacterial use are as follows: (1) the proportion of prescriptions for antibacterial drugs among outpatients, emergency patients, and inpatients (number of prescriptions containing antibacterial agents/total number of prescriptions among outpatients, emergency patients, and inpatients × 100%); (2) the intensity of antimicrobial consumption (total consumption of antibiotics/patient bed-day at the same time × 100); total consumption of antibiotics refers to total the defined daily dose (DDD), which is the average daily dose of adult drugs for primary treatment purposes and is equal to the consumption of an antibacterial drug divided by the DDD; (3) the rate of prophylactic antibacterial agents used during clean surgery (number of prophylactic antibacterial agents used during clean surgery/number of clean surgeries × 100%); (4) the drug selection qualification rate (number of antibacterial agents selected from the drug catalog of prophylactic antibiotic agents/number of prophylactic antibacterials used during clean surgery × 100%); (5) the reasonable rate of antibiotic administration at the time of the first incision (number of reasonable antibiotic agents administered at the time of the first incision/number of prophylactic antibiotic agents used during clean surgery × 100%); (6) reasonable rate of prophylactic treatment with antibiotic agents during clean surgery (number of reasonable treatments with prophylactic antibiotic agents/number of prophylactic antibiotic agents used during clean surgery × 100%)

### National and local guidelines

Antibacterial agents were identified according to the WHO Anatomical Therapeutic Chemical (WHO/ATC) Classification System [22]. According to the standards of the “Guiding Principles of the Clinical Application of Antibacterials” issued by the Chinese government in 2015 [23], the number of antibiotics procured are restricted to 35 or 50 in secondary or tertiary hospitals, respectively. The standards also require that the antibiotic prescription rate should be limited to 60%, 20%, and 40% of all prescriptions for hospitalized patients, outpatients, and emergency patients, respectively; the preventive use rate of antibiotics in clean surgery should be reduced to 30% or below; and the intensity of antibiotic use should be less than 40 daily defined doses (DDD) per 100 patient days.

### Statistical analysis

All collected data were double-entered using Excel 2007 to confirm the accuracy and conduct the analysis. Continuous variables were represented by means and ranges, and categorical data were expressed in proportions. Income and expenses were expressed in US dollars: 1 USD = 6.39 RMB.

An evaluation team was formed to judge the rational use of antimicrobial agents according to the Chinese “Guiding Principles of the Clinical Application of Antibacterials” of 2015 [23]. This team was composed of two clinical pharmacists who specialized in anti-infection, one attending physician who specialized in infectious diseases, and one attending physician who specialized in respiratory diseases.

### Ethical approval

Xi’an Jiaotong University Health Science Center, Shaanxi Provincial Department of Health, the Shaanxi Food and Drug Administration, and all participating hospitals approved the study prior to data collection. The dean of each hospital gave verbal consent for participation in this survey. The requirement for patient consent was waived because there was no contact with patients and patient information was anonymous. Ethical approval for this study was obtained from the Xi’an Jiaotong University Research Ethics Committee.

## Results

### Characteristics of institutions

The general characteristics of all 16 participating institutions are illustrated in Table 1. The total number of patients per year in public tertiary hospitals was slightly higher than those in private tertiary hospitals; however, in secondary hospitals, the opposite result was found. The medical staff primarily consisted of physicians, physician assistants, pharmacists, and nurses. In tertiary hospitals, the proportion of total income from drug sales was 39.2%, with antibiotic sales accounting for 18.2% of this income. In secondary hospitals, the proportion of total income from drug sales was 38.7%, with antibiotic sales accounting for 14.5% of this income.

**Table 1:** General characteristics of hospitals.

Indicators	Tertiary HospitalMean (Range)			Secondary Hospital Mean (Range)		
	Public	Private	All	Public	Private	All
Beds, No.	1000	800	900	185(100–316)	280(100–650)	233(100–650)
Total number of patients per year, No.	637484 (602776–665332)	635745 (600264–657656)	636614 (600264–665332)	98346 (35457–164240)	154067 (37762–407362)	126206 (35457–407362)
Inpatients per year, No.	32113 (31093–32670)	30953 (28349–32949)	31533 (28349–32949)	4623 (648–12450)	12283 (878–34737)	8453 (648–34737)
Prescribers, No.	523(486–557)	335(316–353)	429(316–557)	62(30–107)	102(32–285)	82(30–285)
Nursing staff, No.	711(690–739)	538(500–558)	625(500–739)	100(31–189)	166(41–483)	133(31–483)
Pharmacy staff, No.	64(61–66)	24(22–27)	44(22–66)	14(8–22)	12(6–29)	13(6–29)
Drug income as a percentage of total income, %	47.30 (47.10–47.60)	31.00 (30.00–32.80)	39.20 (30.00–47.60)	43.70 (25.60–58.00)	33.70 (20.90–43.20)	38.70 (20.90–58.00)
Antibiotic sales as a percentage of drug income, %	18.50 (16.40–19.90)	17.80 (16.60–18.70)	18.20 (16.40–19.90)	13.70 (4.90–18.00)	15.30 (6.30–25.30)	14.50 (4.90–25.30)

Analysis of the amount of antibacterial drugs used and the purchase amount found that all types of antibiotics were used. The most commonly used antibiotics were penicillins, cephalosporins, imidazoles, macrolides and fluoroquinolones (Table 2). Whether in tertiary or secondary hospitals, the highest use of antibacterial drugs in public hospitals was penicillin and in private hospitals was cephalosporin. The order of the amount of antibiotics used was the same in the tertiary and secondary hospitals (Table 3).

**Table 2:** Most commonly prescribed antibiotic classes in hospitals.

ATC Code	Antibiotic Class	Antibiotic Sales, g			
		Tertiary Hospital		Secondary Hospital	
		Public	Private	Public	Private
J01CA/E/F	Penicillins	13563341.96	702792.00	1128799.82	1202204.83
J01DA	Cephalosporins	882636.35	1674572.00	1502864.00	2362429.02
J01XD	Imidazoles	90443.73	528735.00	833300.48	3536691.28
J01FA	Macrolides	64783.98	448579.00	206332.13	131111.10
J01MA	Fluoroquinolones	57498.00	162903.00	167534.33	230708.6

**Table 3:** Highest amount of antibiotic classes used in hospitals.

ATC Code	Antibiotic Class	Antibiotic Sales Amount, $			
		Tertiary Hospital		Secondary Hospital	
		Public	Private	Public	Private
J01DA	Cephalosporins	6767190.00	5609697.00	3695161.00	6275265.00
J01CA/E/F	Penicillins	1121453.00	949295.60	1139481.00	1957685.00
J01MA	Fluoroquinolones	993839.10	612623.10	772403.00	1612454.00
J01XD	Imidazoles	451215.60	188182.20	66891.52.00	951813.4.00
J01FA	Macrolides	177056.00	526896.30	253853.60	581196.4.00

### Drug prescribing indicators

As shown in Table 4, the percentage of injectable prescriptions was 14.2% in tertiary hospitals, which was lower than the 26.6% in secondary hospitals, and it was 19.7% in private tertiary hospitals, which was far more than the 8.8% in public tertiary hospitals. The percentage of essential medications among the total amount of drugs was 37.9% in tertiary and 61.5% in secondary hospitals. The rate of essential medications in public institutions was lower than that in private institutions, no matter whether in secondary or tertiary hospitals. The average number of drugs per prescription was 2.36 in secondary hospitals, which was slightly higher than that in tertiary hospitals. The average cost per prescription was 22.6 USD in tertiary hospitals, which was higher than the cost in secondary hospitals. The average cost per prescription was nearly the same in public and private secondary hospitals.

**Table 4:** Indicators of drug prescribing in different levels of hospitals.

Indicators	Tertiary Hospital			Secondary Hospital		
	Public	Private	All	Public	Private	All
Drugs prescribed per visit, mean (range)	1.82(1.55–2.02)	2.31(2.22–2.37)	2.07(1.55–2.37)	2.42(1.10–3.08)	2.30(1.28–3.47)	2.36 (1.10–3.47)
Cost per visit, mean (range), $	22.20 (20.37–23.75)	23.00 (21.10–25.17)	22.60 (20.37–25.17)	15.30 (11.28–30.92)	15.10 (9.78–21.95)	15.20 (9.78–30.92)
Percentage of injections, %	8.80	19.70	14.20	26.90	26.60	26.60
Percentage of essential drugs, %	32.90	42.90	37.90	60.20	63.10	61.50

### Antibiotic use in different levels of hospitals

The results of antibiotic use are shown in Table 5. The number of antibiotic agents procured was 48 in tertiary and 30 in secondary hospitals. In tertiary hospitals, the average percentage of antibiotics prescribed for outpatients, emergency patients, and inpatients was 17.0%, 28.2%, and 54.6%, respectively. The average percentage of antibiotics prescribed for outpatients, emergency patients, and inpatients in secondary hospitals was 20.8%, 31.1%, and 52.2%, respectively. The percentage of antibiotics prescribed for outpatients and emergency patients was higher in private tertiary hospitals than in public tertiary hospitals. In contrast, the percentage of antibiotics prescribed for outpatients was higher in public secondary hospitals than in private secondary institutions. The intensity of antibiotic use was 46.6 DDD/100 bed-days and 48.1 DDD/100 bed-days in tertiary and secondary hospitals, respectively; this was slightly higher in public institutions than in private ones.

**Table 5:** Indicators of drug prescribing in different levels of hospitals.

Indicators	Tertiary Hospital			Secondary Hospital		
	Public	Private	All	Public	Private	All
Antibiotic agents used, No.	50.00	46.00	48.00	30.00	30.00	30.00
**Outpatients**						
Percentage of antibiotics, %	15.00	19.20	17.10	22.40	19.30	20.80
**Emergency**						
Percentage of antibiotics, %	28.00	28.50	28.20	31.10	31.20	31.10
**Inpatients**						
Percentage of antibiotics, %	58.80	50.40	54.60	49.80	54.50	52.20
DDD/100 bed-days	54.10	39.10	46.60	49.00	47.20	48.10

### Antibiotic use in Class I incision surgery

Table 6 shows the antibiotic prophylaxis rates in clean surgery for different levels of hospitals. The preventive use rate of antibiotics was 40.4% in tertiary and 60.7% in secondary hospitals, which were both higher than the national standard of 30%. The preventive use rate of antibiotics in private hospitals was higher than in public institutions. Of all the antibiotic prescriptions for clean surgery patients, the reasonable rate of different antibiotics, proper rate of medication time, and rational rate of medication course in tertiary hospitals were higher than those in secondary hospitals. In addition, the rational rate of medication course was only 66.9% in secondary hospitals, which was far lower than that in tertiary hospitals.

**Table 6:** Antibiotic use in Class I incision surgery.

Indicators	Tertiary Hospital			Secondary Hospital		
	Public	Private	All	Public	Private	All
Antibiotic prophylaxis rates (%)	25.90	55.00	40.40	59.70	61.50	60.70
Proper antibiotic varieties of prophylaxis (%)	100.00	96.10	98.00	79.90	81.70	81.10
Proper medication time of prophylaxis (%)	100.00	99.90	100.00	82.00	81.00	82.20
Proper medication course of prophylaxis (%)	95.10	86.30	90.70	63.80	68.60	66.90

### Antibiotic use in different years

**Fig 1:**
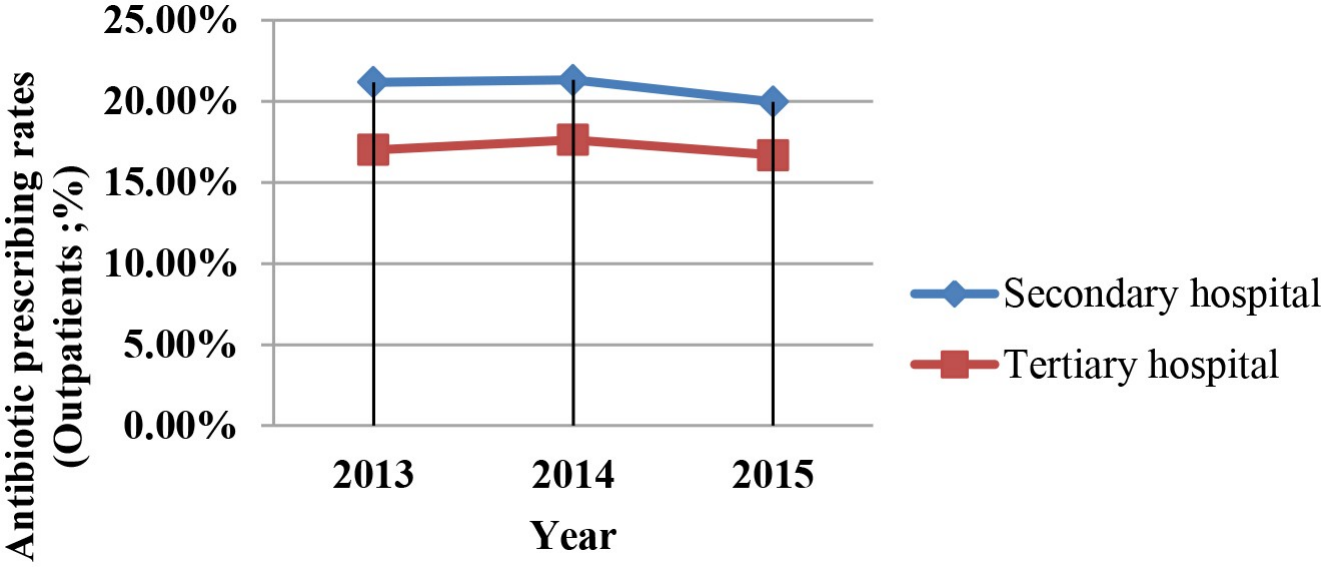
Antibiotic prescription rates of outpatients in tertiary and secondary hospitals. Antibiotic prescription rates of outpatients in Different Levels of Hospitals by Year.

**Fig 2:**
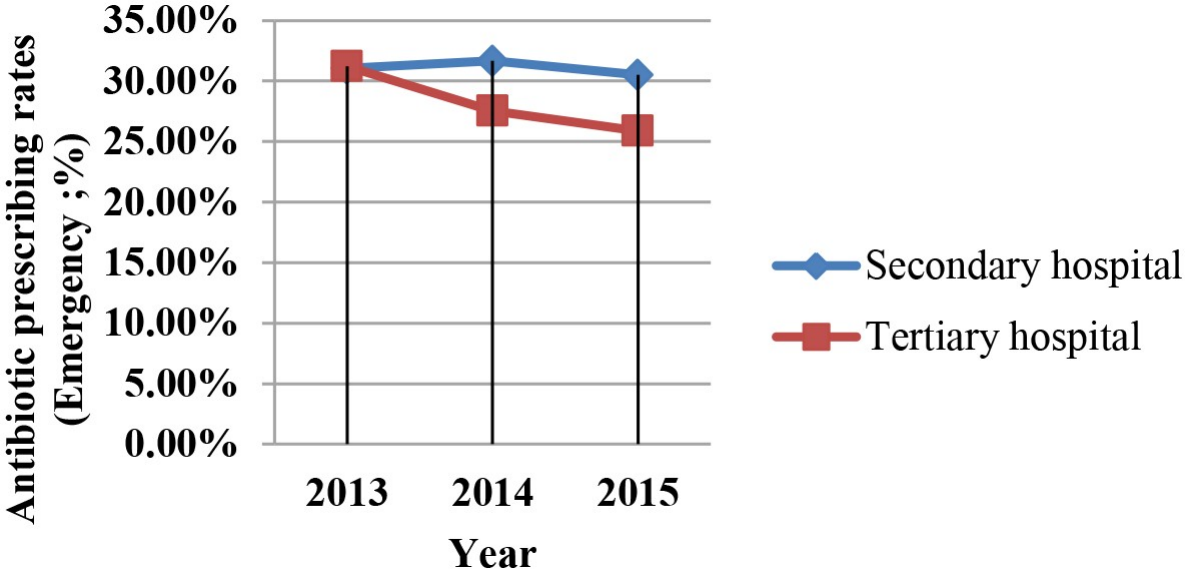
Antibiotic prescription rates of emergency patients in tertiary and secondary hospitals. Antibiotic prescription rates of emergency patients in Different Levels of Hospitals by Year.

**Fig 3:**
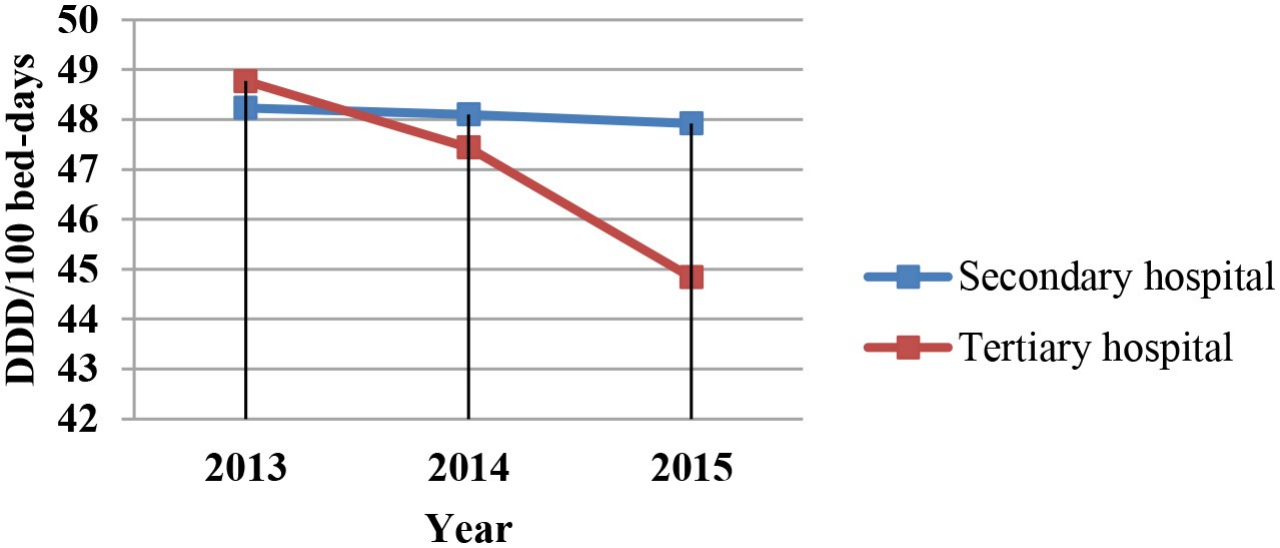
DDD per 100 patient days in tertiary and secondary hospitals. DDD per 100 patient days in Different Levels of Hospitals by Year.

Analysis of the use of antimicrobials in hospitals in different years revealed that DDD per 100 patient days and antibiotic prescription rates for outpatients and emergency patients decreased year by year (Figs 1–3). However, this downward trend varied among the different levels of hospitals. The fastest decline in DDD per 100 patient days during the 3 years was in tertiary hospitals.

## Discussion

As far as we know, this is the first study to report detailed information about the antibiotic prescribing patterns of public and private hospitals in western China after the implementation of the Action Initiative. Our sample included 16 institutions; 8 public sector hospitals and 8 private ones. This study showed that the reasonable use of antibiotics in tertiary hospitals was superior to that of secondary hospitals. In this survey of Chinese hospitals, antibiotic sales as a percentage of income from drug sales was higher in tertiary hospitals than in secondary hospitals; there was no obvious difference between the public and private sectors. A study of Chinese general hospitals found that antibiotics accounted for approximately 17% to 25% of all drug sales [24], which is slightly higher than our findings. This may be because of differences in time frames and geographical areas between the two studies, or different results regarding the implementation effect of the Action Initiative.

In both tertiary and secondary hospitals, the percentage of antibiotics prescribed for outpatients, emergency patients, and inpatients was within the scope of the national standards. Similar to the results of other published research [25–26], the excessive and improper use of antibiotics has been gradually improving, especially after the Action Initiative began. The percentage of antibiotics prescribed for inpatients in tertiary hospitals decreased from 61.4% to 43.8% after the implementation of the Action Initiative [25].

We found that from 2013 to 2015, the rate of antibiotics prescribed for outpatients and emergency patients declined each year, with this rate decreasing more rapidly in tertiary than in secondary hospitals; this clearly demonstrates the sustained effectiveness of this campaign. We also found that there was no significant difference in antibiotic use indicators between public and private hospitals. These results show an effect of the level of the hospital, whereas the type of hospital had no effect. This may be because in China, all hospitals are graded, and requests for the use of antibacterial drugs in the criteria are the same for public and private hospitals.

Regardless of whether in a secondary or tertiary hospital, the intensity of antibiotic use in this study was higher than the national standard of 40 DDD/100 bed-days, which differs slightly from the results of previous research. A study conducted among 65 general hospitals in China found that after the implementation of the Action Initiative, the intensity of antibiotic consumption was 35.9 DDD/100 bed-days [18]. A study from three wards in the largest tertiary teaching hospital in Ethiopia found that three out of four patients were prescribed antibiotics, and the mean antibiotic consumption was 81.6 DDD/100 bed-days [27]. This difference may be owing to a difference in geographic location. From 2013 to 2015, the intensity of antibiotic use decreased more rapidly each year in tertiary hospitals than in secondary hospitals. This may be because the stewardship programs/policies, support staff, etc. were different between tertiary and secondary hospitals.

Regarding clean surgery, only the antibiotic prophylaxis rate in public tertiary hospitals was below the national maximum limit of 30%. In secondary hospitals, the antibiotic prophylaxis rate was very high and the reasonable use rate was unacceptable. Compared with other countries, the rate of reasonable antibiotic use in secondary hospitals in China remains relatively high. An international survey of antimicrobial prophylaxis in surgery found that 26.1% of patients did not adhere to the proper duration of medication administration and 27.2% did not follow the proper medication course [28]. In a survey conducted in Italy from 2009 to 2012, the preoperative antibiotic prophylaxis was appropriate in 18.1% of cases, and the appropriateness of timing of the prophylactic antibiotic administration was observed in 53.4% of procedures [29]. A study conducted in China in 2013 found that the antibiotic prophylaxis rate in Class I incision surgery decreased from 77.38% to 46.60% [30], but it was still very high.

There are many reasons for antibiotics abuse [31–35]. One is the cause of the patient, who may view antibiotics as a panacea, and therefore demand them even when they are unwarranted, or they may demand intravenous over oral therapies, perceiving them to be more efficacious. The other is the supply side; physicians may overprescribe antibiotics because they lack professional knowledge about proper antibiotic usage, or simply because they believe that is what patient wants. This study aimed to explore how antibiotics were used in secondary and tertiary hospitals after the implementation of the Action Initiative.

There are several limitations in this study. First, this research was conducted in one city in China: Xi’an; therefore, the results may not reflect the antibiotic use in hospitals throughout the nation. However, in this pilot study, we used a multistratified grouping random sampling method to reveal the antimicrobial drug use between different levels and different types of hospitals. Second, we did not investigate the characteristics of the medical staff in research hospitals, such as education level, work experience, salary and so on. Finally, the current study revealed the use of antibiotics among outpatients, emergency patients, and inpatients, but we did not analyze the rational use rate of antibiotics by analyzing each prescription. However, we compared the difference between our study results and the national standards, which were designed by policy-makers who considered the rational use of antibiotics before formulating the standards.

## Conclusions

In this study, we found substantial antibiotic overuse in Chinese hospitals. To counter the current situation of antibiotic misuse, policy-makers should continue to improve the administration of antimicrobial use in hospitals. Strict norms are also needed to regulate the prescribing behavior of physicians. At the same time, training of medical professionals and interventions for physicians should be conducted to fundamentally reduce the irrational use of antibiotics, especially in secondary hospitals.

## Supporting information

S1 File Hospital characteristics. General characteristics of hospitals.
(ZIP)

S2 File Antibacterial drug sales. The antibacterial drug sales in different level of hospitals.
(ZIP)

S3 File Indicators of drug prescribing. The indicators of drug prescribing in different level of hospitals.
(ZIP)
